# Up-regulation of heme oxygenase-1 expression and inhibition of disease-associated features by cannabidiol in vascular smooth muscle cells

**DOI:** 10.18632/oncotarget.26191

**Published:** 2018-10-02

**Authors:** Margit Schwartz, Sabine Böckmann, Burkhard Hinz

**Affiliations:** ^1^ Institute of Pharmacology and Toxicology, Rostock University Medical Center, Rostock, Germany

**Keywords:** cannabidiol, smooth muscle cells, heme oxygenase-1, proliferation, migration

## Abstract

Aberrant proliferation and migration of vascular smooth muscle cells (VSMC) have been closely linked to the development and progression of cardiovascular and cancer diseases. The cytoprotective enzyme heme oxygenase-1 (HO-1) has been shown to mediate anti-proliferative and anti-migratory effects in VSMC. This study investigates the effect of cannabidiol (CBD), a non-psychoactive cannabinoid, on HO-1 expression and disease-associated functions of human umbilical artery smooth muscle cells (HUASMC). HO-1 protein and mRNA were significantly increased by CBD in a time- and concentration-dependent manner. Although the expression of several cannabinoid-activated receptors (CB_1_, CB_2_, G protein-coupled receptor 55, transient receptor potential vanilloid 1) was verified in HUASMC, CBD was shown to induce HO-1 via none of these targets. Instead, the CBD-mediated increase in HO-1 protein was reversed by the glutathione precursor N-acetylcysteine, indicating the participation of reactive oxygen species (ROS) signaling; this was confirmed by flow cytometry-based ROS detection. CBD-induced HO-1 expression was accompanied by inhibition of growth factor-mediated proliferation and migration of HUASMC. However, neither inhibition of HO-1 activity nor knockdown of HO-1 protein attenuated CBD-mediated anti-proliferative and anti-migratory effects. Indeed, inhibition or depletion of HO-1 resulted in induction of apoptosis and intensified CBD-mediated effects on proliferation and migration. Collectively, this work provides the first indication of CBD-mediated enhancement of HO-1 in VSMC and potential protective effects against aberrant VSMC proliferation and migration. On the other hand, our data argue against a role of HO-1 in CBD-mediated inhibition of proliferation and migration while substantiating its anti-apoptotic role in oxidative stress-mediated cell fate.

## INTRODUCTION

Atherosclerosis, formerly considered a response to endothelial injury [1], is an inflammatory disease provoked by multifactorial-triggered endothelial dysfunction, with vascular smooth muscle cells (VSMC) being of great importance in the progression of this process [2–4]. Importantly, aberrant proliferation and migration of VSMC have been linked to several pathological events such as intimal hyperplasia [5], in-stent restenosis [6, 7] and blood vessel formation during cancer and other non-neoplastic disorders [8, 9]. VSMC are not terminally differentiated and thus possess the ability to undergo complex regulated phenotype switching [10, 11]. At the physiological level, VSMC predominantly present a contractile/quiescent phenotype and are substantially involved in the regulation of blood pressure and vascular tone [12]. However, growth factor signaling or oxidative stress mediate the transition of VSMC towards a proliferative/synthetic phenotype, thereby conferring synthesis of extracellular matrix proteins and enhanced proliferation and migration [4, 11]. Attenuation of abnormal VSMC proliferation and migration could therefore be a useful pharmacotherapeutic strategy to prevent processes promoting the development and the progress of vascular [4, 7, 13, 14] and cancer diseases [9].

The cytoprotective heme oxygenase (HO) system, consisting of an inducible HO-1 and a constitutive HO-2 isoform [15], has attracted considerable attention in recent years in the context of vascular diseases (reviewed in [16, 17]). HO enzymes regulate cellular heme homeostasis via rate limitation of oxidative heme breakdown [15, 18], thereby removing the potential cytotoxic molecule heme [19–21] and producing equimolar amounts of biliverdin and carbon monoxide; both are potential cytoprotective substances [22–24]. As a matter of fact, numerous investigations published so far reported a pivotal role of HO-1 in protection against disease-associated features of VSMC such as proliferation [25–32] and migration [26–29]. Additionally, some reports have documented that HO-1 is beneficial against oxidative stress [30, 33] and inflammatory responses [25] in VSMC.

In recent years, cannabinoids have been increasingly the focus in medical research as potential pharmacotherapeutic approaches for diverse indications [34–37]. The phytocannabinoid cannabidiol (CBD), which lacks psychoactivity in contrast to Δ^9^-tetrahydrocannabinol (THC), has emerged as a preferable substance for preclinical research [38]. CBD is a component of nabiximols, a standardized cannabis extract containing roughly equal amounts of CBD and THC, which has been approved for the treatment of multiple sclerosis-associated spasticity by the European Medicines Agency as well as for the treatment of neuropathic multiple sclerosis-associated and opioid-resistant cancer-related pain by Health Canada [39]. Previous studies by our group have shown the potential anti-cancer effects of CBD [40–44]. Others have demonstrated the anti-oxidant properties of CBD, conferring a reduction in lipopolysaccharide-induced vascular inflammation in the mouse brain [45], protection against hepatic ischemia-reperfusion injury [46] and acute alcohol-induced liver steatosis [47] in mouse models as well as reduced endoplasmic reticulum stress in oligodendrocyte progenitor cells [48].

Moreover, and in the context of vascular diseases, the cardiovascular system was described to be a potential target for CBD. Accordingly, CBD has been shown to attenuate the high glucose-induced inflammatory response and barrier disruption in human coronary artery endothelial cells, indicating potential benefits against diabetic complications and atherosclerosis [49]. The cardioprotective effects of CBD were further demonstrated *in vivo* using a rat ischemia-reperfusion model [50] and a mouse model of diabetic cardiomyopathy, where CBD attenuated myocardial dysfunction via a reduction in cardiac fibrosis, oxidative/nitrative stress, inflammation and cell death [51]. Independent of its diverse protective actions, the impact of CBD on disease-associated features of VSMC, particularly proliferation and migration, and HO-1 expression has not been addressed so far. Using human umbilical artery smooth muscle cells (HUASMC), the present study demonstrates favorable anti-proliferative and anti-migratory effects of CBD in VSMC for the first time, along with a profound induction of the cytoprotective enzyme HO-1.

## RESULTS

### Phytocannabinoids induce HO-1 protein expression in HUASMC

**Figure 1:**
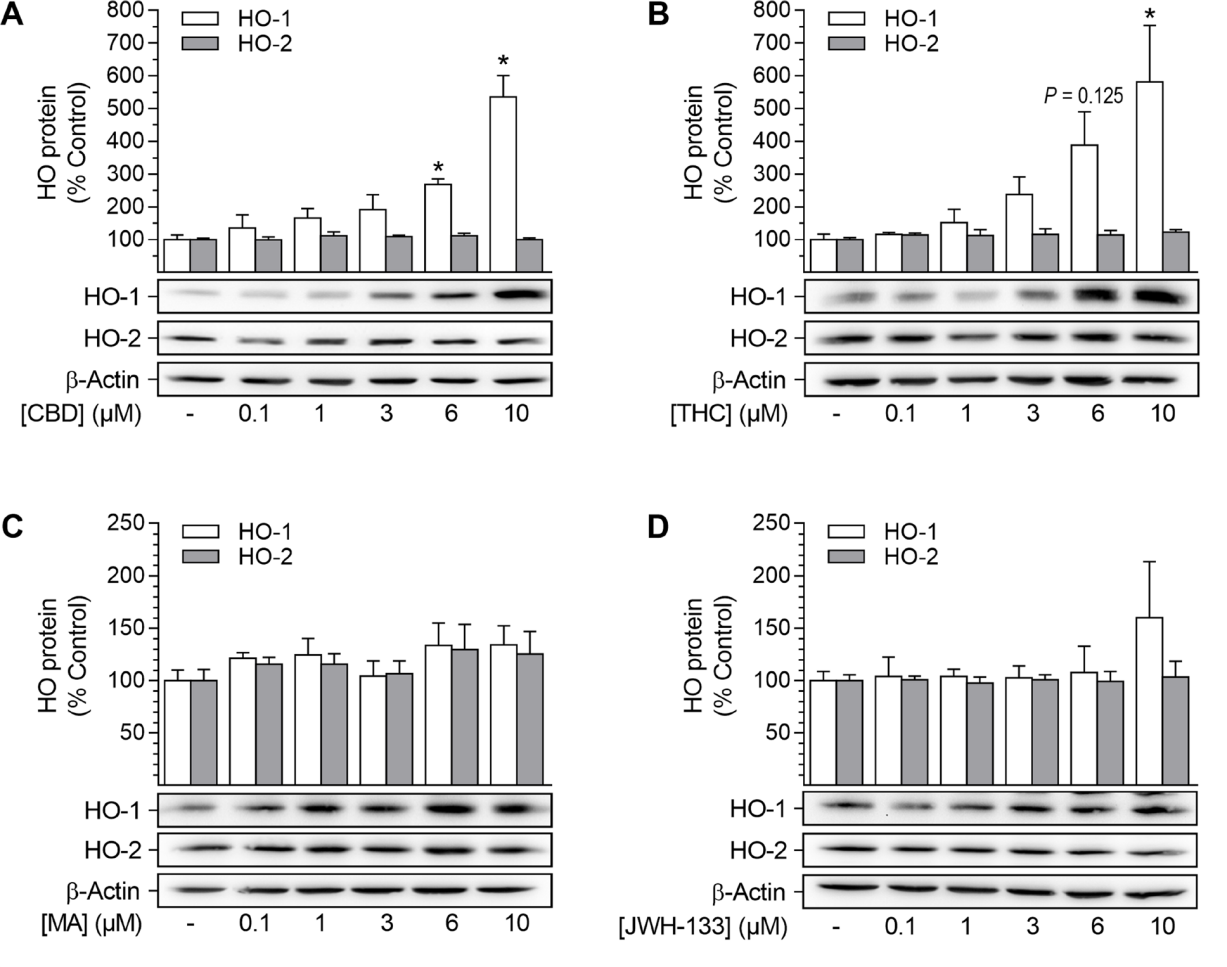
Effect of cannabinoids on HO-1 and HO-2 protein expression in HUASMC Cells were incubated for 24 h with CBD (**A**), THC (**B**), R(+)-methanandamide (MA) (**C**) or JWH-133 (**D**) at the indicated concentrations. Following incubation, cells were harvested and lysates were analyzed for protein expression of HO-1 and HO-2. Protein expression values were normalized to β-actin. Percentage of control represents comparison with the respective vehicle-treated time-matched group (set as 100%), according to densitometric analysis. Western blot images are representative of each experiment. Values are means ± SEM of *n =* 4 (A, HO-1), *n =* 5 (A, HO-2) or *n =* 3 (B, C, D) experiments. ^*^P < 0.05 vs. time-matched vehicle control; one-way ANOVA plus Dunnett post hoc test.

In a first experimental approach, four different cannabinoids, i.e. the phytocannabinoids CBD and THC (CB_1_/CB_2_ agonist), as well as the synthetic cannabinoids R(+)-methanandamide (CB_1_ agonist) and JWH-133 (CB_2_ agonist), were analyzed for their potential to induce the expression of HO-1 in HUASMC (Figure 1). Both CBD and THC significantly increased HO-1 protein expression in a concentration-dependent manner after a 24-h incubation period (Figure 1A, 1B). CBD-mediated induction of HO-1 protein was significant at 6 µM and 10 µM CBD, resulting in 2.7-fold and 5.4-fold increases in HO-1 protein, respectively (Figure 1A). Similarly, the expression of HO-1 protein was significantly increased by 5.8-fold when cells were incubated with 10 µM THC (Figure 1B). Conversely, neither R(+)-methanandamide nor JWH-133 significantly enhanced protein expression of HO-1 (Figure 1C, 1D). Finally, none of the tested cannabinoids altered the protein expression of HO-2 (Figure 1A–1D). Due to its lack of psychoactivity and potent induction of HO-1, CBD appeared to be an interesting candidate substance for therapeutic applications and was therefore selected for further investigations.

### CBD mediates increases of HO-1 mRNA and protein levels in HUASMC in a time-dependent manner

**Figure 2:**
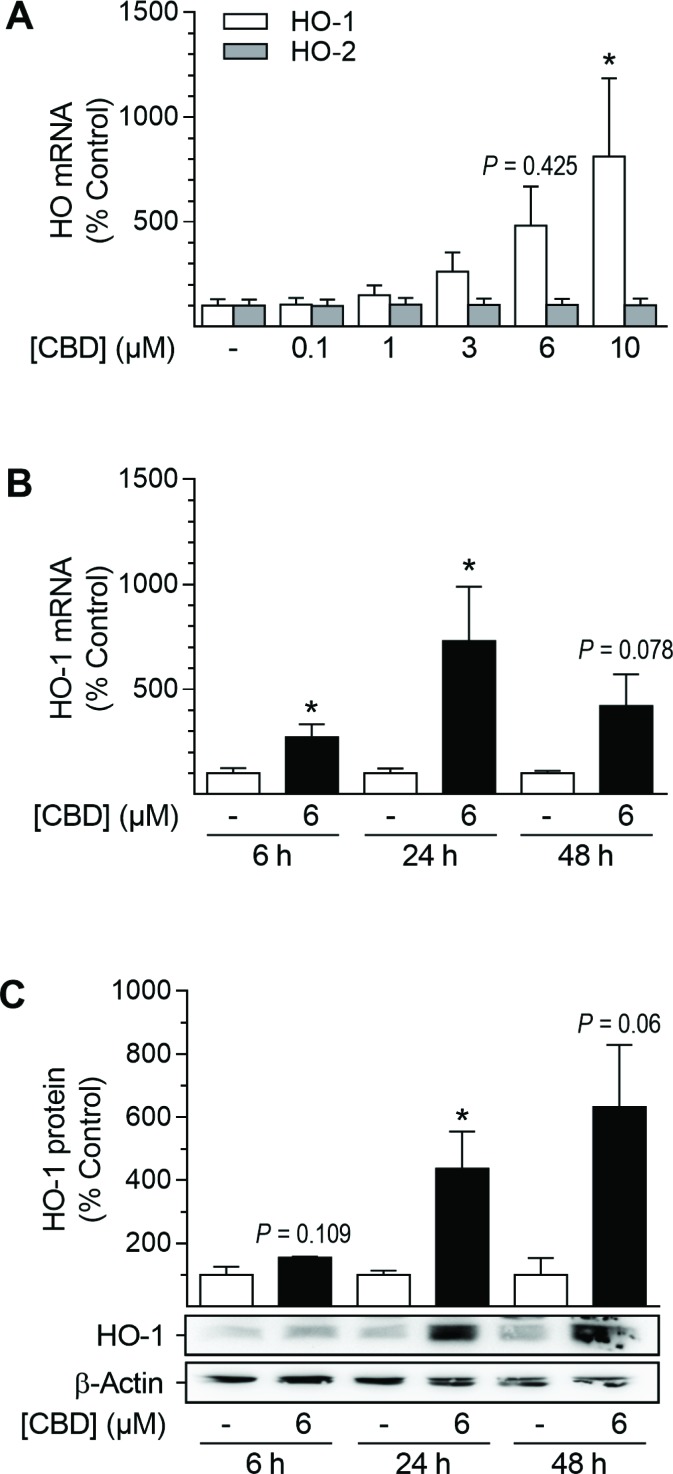
Effect of CBD on HO-1 and HO-2 mRNA and HO-1 protein expression in HUASMC Cells were incubated for 24 h with CBD at the indicated concentrations (**A**) or with 6 µM CBD for the indicated times (**B**, **C**). After incubation, cells were analyzed for mRNA expression of HO-1 (A, B) and HO-2 (A) or protein expression of HO-1 (C). Expression values were normalized to β-actin. Percentage of control represents comparison with the respective vehicle-treated time-matched group (set as 100%). Western blot images are representative of each experiment. Values are means ± SEM of *n =* 6 (A), *n =* 3–5 (B) or *n =* 3 (C) experiments. ^*^P < 0.05 vs. time-matched vehicle control; one-way ANOVA plus Dunnett post hoc test (A) or Student’s two-tailed *t*-test (B, C).

Analyses regarding the involvement of mRNA expression and kinetic experiments were performed to further characterize CBD-mediated HO-1 induction (Figure 2). HO-1 mRNA expression was significantly enhanced after incubation with 10 µM CBD for 24 h (Figure 2A). Kinetic studies revealed the CBD-mediated induction of HO-1 mRNA to be time-dependent: enhancement of mRNA expression was significant after 6 h (2.7-fold increase), peaked after 24 h with a 7.3-fold increase and then declined during 48 h of incubation with 6 µM CBD (Figure 2B). However, mRNA levels of HO-2 were not altered by CBD at any concentration tested (Figure 2A) or during kinetic experiments (data not shown). According to the obtained mRNA data, HO-1 protein expression progressively increased up to 6.3-fold during a 48-h incubation period with 6 µM CBD (Figure 2C).

### CB_1_, CB_2_, TRPV1 and GPR55 are expressed in HUASMC, but do not contribute to CBD-mediated HO-1 induction

**Figure 3:**
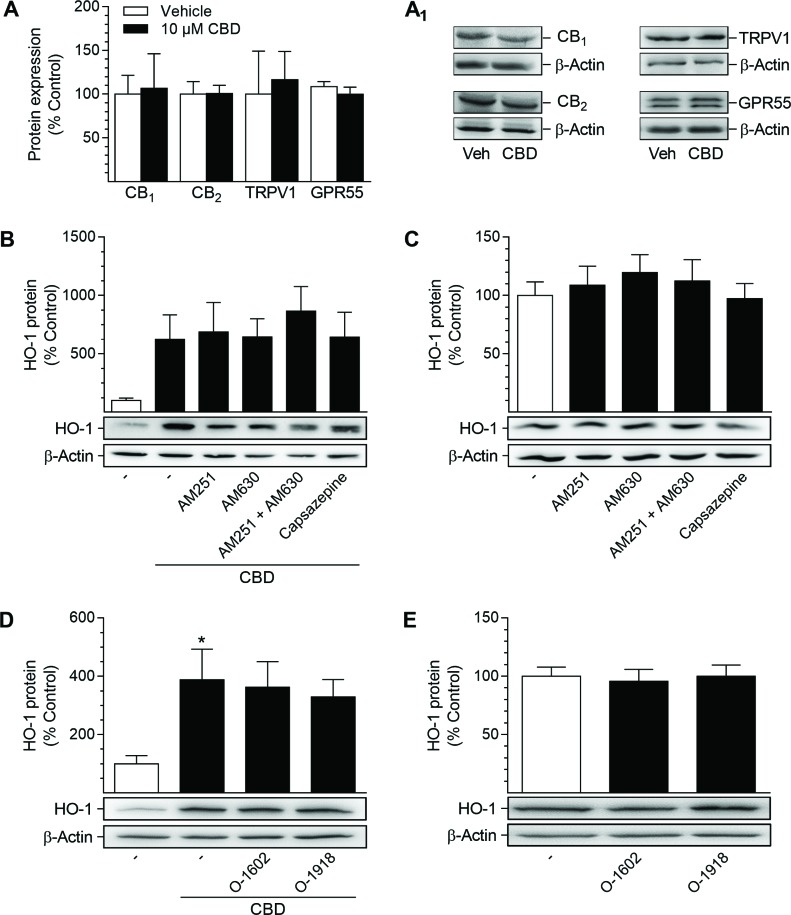
Presence of potential target receptors of CBD in HUASMC and investigation of their involvement in CBD-mediated induction of HO-1 protein For analysis of target receptor presence, HUASMC were incubated with 10 µM CBD or vehicle for 24 h (**A**, **A**_**1**_). For investigation of receptor involvement, HUASMC were pre-incubated with AM251 (CB_1_ antagonist), AM630 (CB_2_ antagonist), capsazepine (TRPV1 antagonist), O-1602 (GPR55 agonist) or O-1918 (GPR55 antagonist) at respective concentrations of 1 µM for 1 h and then further co-incubated with (**B**, **D**) or without (**C**, **E**) 10 µM CBD for 24 h. Following incubation, cells were harvested and lysates were analyzed for protein expression of targeted receptors (A, A_1_) or HO-1 (B–E). Protein expression values were normalized to β-actin. Percentage of control represents comparison with the respective vehicle-treated time-matched group (set as 100%), according to densitometric analysis. Western blot images are representative of each experiment. Values are means ± SEM of *n =* 3 (A), *n* = 5 (B, C, D) or *n =* 6 (E) experiments. ^*^P < 0.05 vs. vehicle-treated group; one-way ANOVA plus Šidák post hoc test. Statistical analysis performed by Student’s two-tailed *t*-test (A), one-way ANOVA plus Šidák post hoc test (B) or one-way ANOVA plus Dunnett post hoc test (C, E), revealed no significant differences between CBD- and vehicle-treated groups (A, B), between cells treated with CBD alone or in combination with receptor activity-modulating substances (B, D) or between vehicle-treated groups and groups treated with receptor activity-modulating substances (C, E).

Next, the potential involvement of cannabinoid-activated receptors in the CBD-mediated induction of HO-1 was investigated (Figure 3). To this end, HUASMC, treated with 10 µM CBD or the respective vehicle, were analyzed first for the presence of CB_1_, CB_2_, G protein-coupled receptor 55 (GPR55) and the non-selective cation channel transient receptor potential vanilloid 1 (TRPV1). All of the mentioned targets were expressed by HUASMC and were neither increased nor decreased after 24 h of incubation with CBD, as shown by Western blot analysis (Figure 3A, 3A_1_). To further investigate the involvement of these receptors in CBD-mediated HO-1 induction, receptor signaling was inhibited by antagonists targeting CB_1_ (AM251), CB_2_ (AM630), TRPV1 (capsazepine) and GPR55 (O-1918) prior to stimulation with CBD (Figure 3B–3E). Additionally, a potential antagonistic effect of CBD on GPR55 was investigated using the GPR55 agonist O-1602 (Figure 3D–3E). Receptor antagonists and O-1602 were used at a concentration of 1 µM, as this has been shown to sufficiently regulate the respective receptor activity [52–55]. Co-incubation with antagonists targeting CB_1_, CB_2_ and TRPV1 did not alter CBD-mediated induction of HO-1 in HUASMC after 24 h of incubation with 10 µM CBD (Figure 3B), without showing effects on HO-1 protein expression per se (Figure 3C). Similarly, neither O-1908 nor O-1602 altered the CBD-mediated increase in HO-1 protein expression (Figure 3D) or basal HO-1 protein levels (Figure 3E).

### CBD-mediated HO-1 induction in HUASMC depends on reactive oxygen species (ROS)

**Figure 4:**
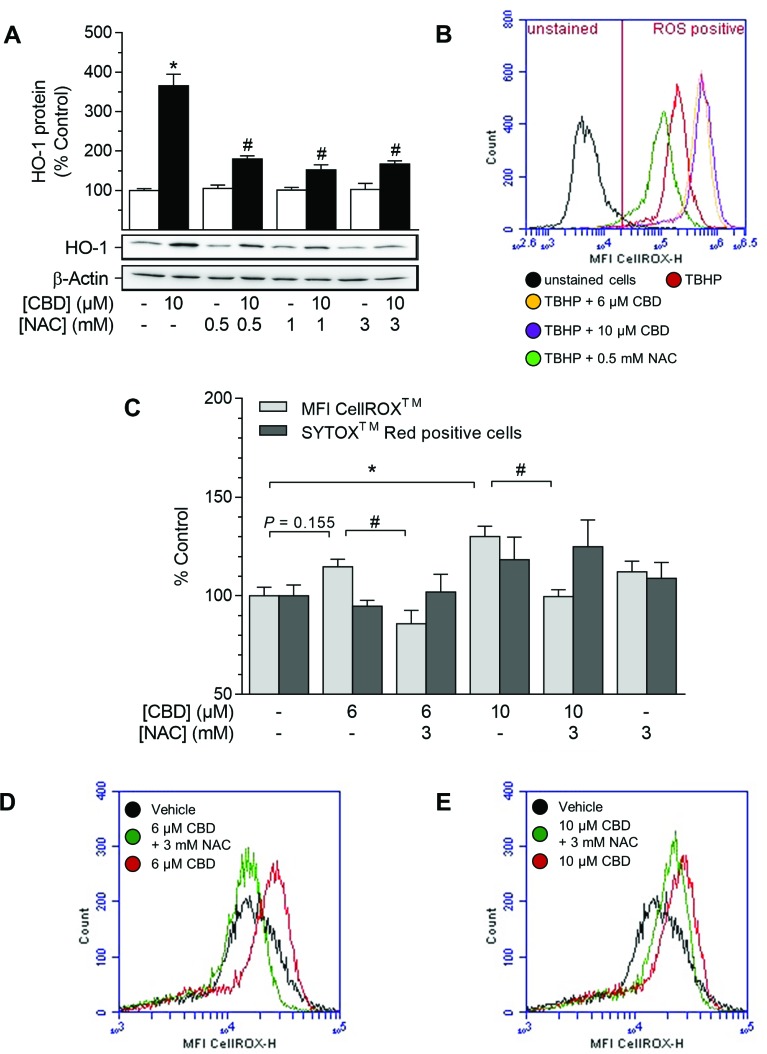
Effect of NAC on CBD-mediated induction of HO-1 and ROS generation in HUASMC Cells were pre-incubated with indicated concentrations of NAC for 1 h and then further co-incubated with indicated concentrations of CBD for 24 h (**A**) or 4 h (**B**–**E**). Protein expression values (A) were normalized to β-actin. Results of an exemplary experiment, investigating the oxidative potential of CBD, are shown in (B). For the detection of ROS generation (B–E), TBHP (250 µM) was added as positive control 1 h prior to sample preparation. Following incubation, cells were harvested and analyzed for protein expression of HO-1 (A) or oxidative stress level using flow cytometry (B–C). MFI overlay diagrams (D, E) are representative of the data shown in C. Percentage of control represents comparison with the respective vehicle-treated time-matched group (set as 100%). Western blot images are representative of the experiment. Values are means ± SEM of *n =* 4 (A) or *n =* 6 (C) experiments. ^*^P < 0.05 vs. time-matched vehicle control; ^#^*P* < 0.05 vs. CBD-treated group; one-way ANOVA plus Šidák post hoc test.

In order to examine the involvement of ROS in CBD-mediated HO-1 induction, the oxidant-scavenging glutathione system was strengthened by pre-incubation with the glutathione precursor N-acetylcysteine (NAC) prior to stimulation with CBD (Figure 4). CBD-mediated HO-1 induction was significantly reduced by NAC at 0.5–3 mM, while NAC did not alter HO-1 protein expression per se (Figure 4A). The pro-oxidant capability of CBD was further substantiated by flow cytometry-based ROS detection using the ROS-sensitive probe CellROX™ Green (Figure 4B–4E). In an exemplary experiment, tert-butyl hydroperoxide (TBHP), an inducer of ROS, strongly increased the mean fluorescence intensity (MFI) of CellROX™-stained HUASMC (Figure 4B, red line), whereby the MFI of CellROX™ in TBHP-treated cells was reduced by pre-incubation with 0.5 mM NAC (Figure 4B, green line). Additionally, TBHP-mediated MFI of CellROX™-stained HUASMC was further increased by pre-incubation with CBD at 6 µM (Figure 4B, yellow line) and 10 µM (Figure 4B, violet line) prior to treatment with TBHP. To support the relationship between CBD-mediated ROS generation and HO-1, we analyzed whether CBD alone increased the MFI of CellROX™ in HUASMC (Figure 4C–4E). The MFI of CellROX™-stained HUASMC was increased by 15% after a 4-h incubation period with CBD at 6 µM (Figure 4C, 4D, red line [exemplary MFI overlay diagram]) and was significantly increased to 130% at 10 µM CBD (Figure 4C, 4D, red line [exemplary MFI overlay diagram). Additionally, the CBD-mediated enhancement in the MFI of CellROX™-stained HUASMC was significantly attenuated by pre-incubation with 3 mM NAC at both 6 µM and 10 µM CBD (Figure 4C–4E, green line). Flow cytometry-based analysis of cell death, which was accomplished in parallel to ROS detection by co-staining with SYTOX™ Red, however, revealed no significant increase in SYTOX™ Red positive cells after treatment with CBD alone or in combination with NAC (Figure 4C).

### HUASMC cultured in complete smooth muscle cell growth medium (cSMCGM) display properties of a proliferative/synthetic phenotype

Dependent on diverse circumstances, VSMC possess the ability to switch between a contractile/quiescent and a proliferative/synthetic phenotype [10, 11]. To classify the phenotype of HUASMC in our experiments, cells were subjected to differently composed media followed by an analysis of proliferation using bromodeoxyuridine (BrdU) incorporation assay (Table 1). The media used here were complete SMCGM (cSMCGM, supplemented with growth factors, 5% fetal calf serum [FCS]) and reduced SMCGM (rSMCGM, no supplements, FCS content as indicated). The proliferation of HUASMC was significantly decreased when cells were incubated under conditions of growth factor deprivation: rSMCGM (5% FCS; 60%) vs. cSMCGM (5% FCS; 100%). Serum reduction further attenuated cell proliferation by 52% and 96% in rSMCGM containing 2% FCS and 0% FCS, respectively, when compared to rSMCGM with 5% FCS. The above-mentioned results suggest that HUASMC incubated in cSMCGM represent a proliferative/synthetic phenotype. Therefore, experiments investigating the effects of CBD on the potential disease-associated features of HUASMC were performed either in cSMCGM (5% FCS) or using a combination of rSMCGM (2% FCS) and treatment with recombinant human platelet-derived growth factor BB (hPDGF-BB [homodimer], subsequently abbreviated as PDGF) as a specific stimulator of the proliferative/synthetic phenotype.

**Table 1:** Effect of medium composition on proliferation of HUASMC

Medium	Serum content	BrdU incorporation (% Control)
cSMCGM	FCS (5%)	100.0 ± 1.1
rSMCGM	FCS (5%)	60 ± 3^*^
rSMCGM	FCS (2%)	29 ± 3^#^
rSMCGM	FCS (0%)	2.4 ± 0.6^#^

Note: Cells were incubated in cSMCGM (containing supplements and 5% FCS) or rSMCGM (no supplements, FCS content as indicated) for 48 h and analyzed for BrdU incorporation. Percent control represents comparison with the respective cSMCGM-incubated time-matched group (set as 100%). Values are means ± SEM of *n =* 8 experiments. ^*^P < 0.05 vs. cSMCGM; ^#^*P* < 0.05 vs. rSMCGM (5% FCS); one-way ANOVA plus Šidák post hoc test.

### CBD triggers inhibition of proliferation without affecting the viability of HUASMC

**Figure 5:**
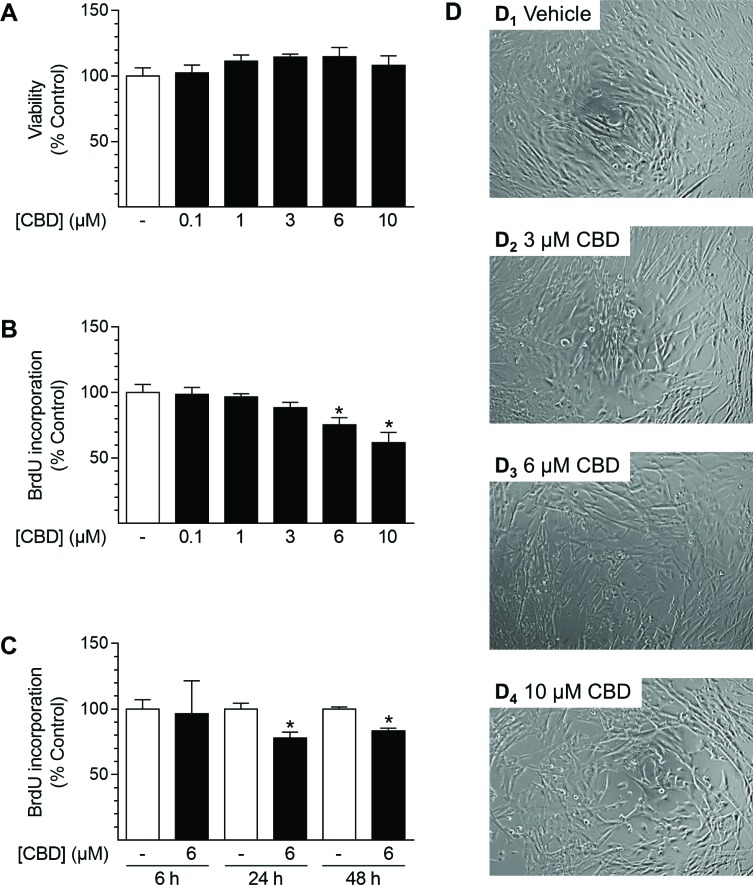
Effect of CBD on viability and proliferation of HUASMC Cells were incubated for 48 h with the indicated concentrations of CBD (**A**, **B**, **D**) or with 6 µM CBD for the indicated times (**C**). Following incubation, cells were analyzed for viability via trypan blue exclusion staining (A) or proliferation using BrdU incorporation assay (B, C). For visualization, unstained monolayers were photographed in culture using a 10× objective and digital microscope camera (AxioCam ERc 5 s; Zeiss) (D). Percentage of control represents comparison with the respective vehicle-treated time-matched group (set as 100%). Values are means ± SEM of *n* = 4 (A) or *n =* 10–12 (B, C) experiments. ^*^P < 0.05 vs. time-matched vehicle control; one-way ANOVA plus Dunnett post hoc test (A, B) or Student’s two-tailed *t*-test (C).

To investigate the effects of CBD on cellular functions, experiments analyzing cell viability and proliferation were performed in cSMCGM (Figure 5). As shown in Figure 5A, the viability of HUASMC, as analyzed by trypan blue staining, was not affected after 48 h of incubation with CBD at any concentration tested (0.1–10 µM). Furthermore, CBD concentration-dependently inhibited proliferation with a significant decrease to 75% and 62% vs. vehicle control (100%) at 6 µM and 10 µM CBD, respectively (Figure 5B). Kinetic studies revealed that proliferation was significantly inhibited after 24 h and 48 h of incubation with 6 µM CBD, with a maximal inhibition of proliferation by 22% after 24 h (Figure 5C). The concentration-dependent anti-proliferative effect was also visible in microphotographs showing the cell densities of the cultured monolayers (Figure 5D). Herein, vehicle-treated cells seeded at sub-confluence at the beginning of the experiment formed a confluent monolayer (Figure 5D_1_), whereas monolayer density did not increase after incubation with increasing concentrations of CBD (Figure 5D_2–4_).

### Anti-proliferative effects of CBD in HUASMC are independent of cannabinoid-activated receptors or ROS signaling

**Figure 6:**
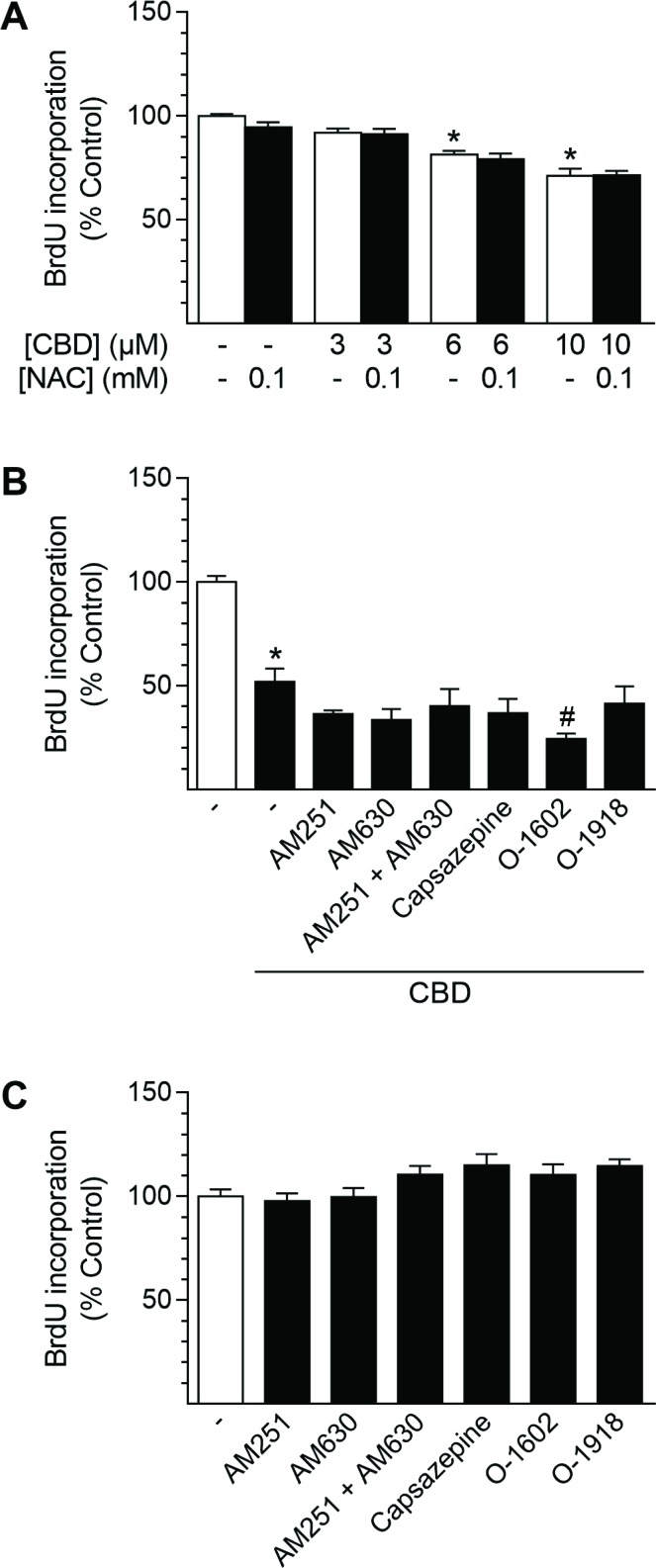
Involvement of ROS generation and receptor signaling in CBD-mediated anti-proliferative effects in HUASMC Cells were pre-incubated with 0.1 mM NAC (**A**) or with 1-µM concentrations of the receptor activity modulating substances AM251 (CB_1_ antagonist), AM630 (CB_2_ antagonist), capsazepine (TRPV1 antagonist), O-1602 (GPR55 agonist) or O-1918 (GPR55 antagonist) (**B**) for 1 h prior to a 24-h co-incubation with CBD as indicated (A) or at 10 µM CBD (B). In another setup (**C**), cells were incubated with receptor activity modulating substances alone for 24 h. Following incubation, cells were analyzed for BrdU incorporation. Percentage of control represents comparison with the respective vehicle-treated time-matched group (set as 100%). Values are means ± SEM of *n* = 11–12 (A), *n =* 10–12 (B) or *n =* 7–8 (C) experiments. ^*^P < 0.05 vs. time-matched vehicle control; ^#^*P* < 0.05 vs. CBD-treated group; one-way ANOVA plus Šidák post hoc test (A, B). Data in (C) were analyzed by one-way ANOVA plus Dunnett post hoc test.

In order to obtain insight into the mechanism by which CBD mediates its anti-proliferative effects in HUASMC, cells were pre-incubated with the glutathione precursor NAC (Figure 6A) or substances targeting CB_1_, CB_2_, TRPV1 or GPR55 prior to treatment with CBD (Figure 6B). Pre-incubation with NAC at 0.1 mM, a concentration that was sufficient to significantly inhibit induction of HO-1 following 24 h of incubation with 10 µM CBD (vehicle control [100% ± 20%] vs. 10 µM CBD [1700% ± 300%], *P* < 0.05; 10 µM CBD vs. 10 µM CBD + NAC [1060% ± 300%], *P* < 0.05; means ± SEM of *n =* 6; one-way ANOVA plus Šidák post hoc test) did not abolish the anti-proliferative effect of CBD, which was significant at concentrations of 6–10 µM CBD (Figure 6A). Herein, incubation with NAC alone did not result in anti-proliferative effects of the substance per se (Figure 6A). Further experiments aimed to investigate the potential involvement of CB_1_, CB_2_, TRPV1 and GPR55 in the CBD-mediated anti-proliferative effects in HUASMC (Figure 6B). Incubation with 10 µM CBD for 24 h significantly reduced HUASMC proliferation to 52% vs. vehicle-treated control (100%). However, pre-incubation with the respective receptor activity modulating substances did not prevent or attenuate this effect (Figure 6B). Indeed, co-incubation with the GPR55 agonist O-1602 further enhanced the anti-proliferative effect of CBD, resulting in 24% BrdU incorporation vs. vehicle-treated control (100%) (Figure 6B). Moreover, none of the receptor activity modulating substances alone significantly altered HUASMC proliferation after a 24-h incubation period (Figure 6C).

### Inhibition of HO-1 activity does not attenuate CBD-mediated effects but induces cell death

**Figure 7:**
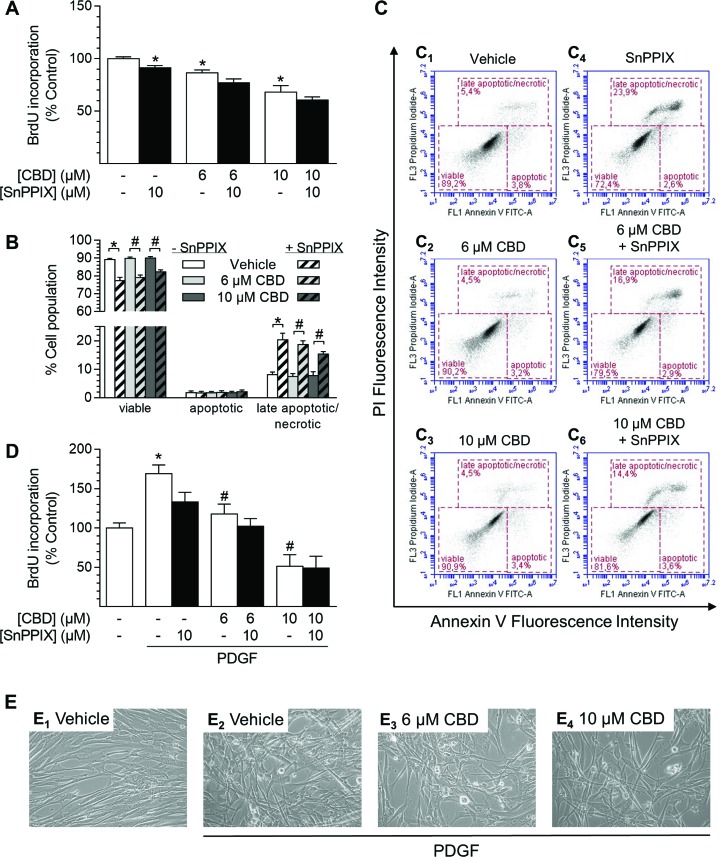
Effect of the HO-1 activity inhibitor SnPPIX on CBD-mediated anti-proliferative effects and viability of HUASMC Cells were pre-incubated with 10 µM SnPPIX for 1 h followed by a 48-h co-incubation period with vehicle or CBD as indicated before the analysis of proliferation (**A**) or apoptosis (**B**). Exemplary images of flow cytometry-based analysis of apoptosis (B) are shown in (**C**). Cells seeded in rSMCGM were pre-incubated with 10 µM SnPPIX for 1 h prior to the addition of CBD as indicated and PDGF (20 ng/ml) or respective vehicles followed by a 24-h co-incubation period before analysis of BrdU incorporation (**D**). For visualization, unstained monolayers were photographed in culture using a 10× objective and digital microscope camera (AxioCam ERc 5 s; Zeiss) (**E**). Percentage of control represents comparison with the respective vehicle-treated time-matched group (set as 100%). Values are means ± SEM of *n* = 10–12 (A, D) or *n =* 8 (B) experiments. ^*^P < 0.05 vs. time-matched (PDGF-free) vehicle control (A, D) or as indicated (B); ^#^*P* < 0.05 vs. PDGF-treated vehicle control (D) or as indicated (B); one-way ANOVA plus Šidák post hoc test.

To address a possible relationship between the anti-proliferative effects of CBD and its impact on HO-1 expression in HUASMC, additional experiments using the HO-1 inhibitor tin protoporphyrin IX (SnPPIX) were performed (Figure 7). The efficiency of SnPPIX in inhibiting HO activity was explained by its competitive antagonism, resulting from blockade of the heme binding site of HO-1 [56]; this has been further demonstrated using bilirubin formation assays in several studies [56–58].

Co-incubation with SnPPIX (10 µM) did not attenuate anti-proliferative effects of CBD (6 µM and 10 µM) after 48 h of incubation (Figure 7A). Indeed, SnPPIX alone significantly decreased HUASMC proliferation (SnPPIX: 87% vs. vehicle control [100%]) and slightly enhanced CBD-mediated anti-proliferative effects (Figure 7A). Flow cytometry-based analysis of apoptosis showed that CBD alone (6 µM and 10 µM) did not decrease cell viability after 48 h (Figure 7B, 7C_1–3_). Conversely, inhibition of HO-1 activity by SnPPIX significantly reduced cell viability and increased the amount of late apoptotic/necrotic cell population when administered alone or in co-incubation with CBD (Figure 7B, 7C_4–6_). However, the proportion of early apoptotic cells, which are positive for annexin V only, was not increased by any treatment (Figure 7B, 7C).

In another experiment we aimed to investigate the role of HO-1 in the impact of CBD on PDGF-mediated proliferation of HUASMC (Figure 7D). Therefore, experiments were conducted in rSMCGM (no supplements, 2% FCS) using 20 ng/ml PDGF, which was shown to sufficiently stimulate HUASMC proliferation under these conditions in a preliminary experiment (Table 2). Cells, pre-treated with SnPPIX, were incubated in the presence of CBD and PDGF or the respective vehicles for 24 h prior to the analysis of BrdU incorporation (Figure 7D). Incubation with PDGF alone significantly enhanced proliferation up to 170% vs. vehicle-treated control (100%), which was also visible by changes in monolayer density and organization compared to vehicle-treated cells (Figure 7E_1–2_). The pro-proliferative effect of PDGF was significantly reduced to 118% and 52% by CBD at 6 µM and 10 µM, respectively (Figure 7D). This anti-proliferative effect was also recognizable in microphotographs, where cell density in the presence of PDGF was considerably reduced by CBD at 6 µM and 10 µM, respectively (Figure 7E_2–4_). Similar to the results in Figure 7A, inhibition of HO-1 activity by SnPPIX alone reduced PDGF-induced proliferation of HUASMC to 133% (Figure 7D). However, co-incubation with SnPPIX neither significantly enhanced nor attenuated the anti-proliferative effects of CBD under these conditions (Figure 7D).

**Table 2:** Effect of PDGF on proliferation of HUASMC

Treatment	BrdU incorporation (% Control)
Vehicle PDGF (10 ng/ml) PDGF (20 ng/ml) PDGF (40 ng/ml) PDGF (60 ng/ml)	100 ± 3 182 ± 12^*^ 196 ± 10^*^ 215 ± 14^*^ 219 ± 14^*^

Note: Cells seeded in rSMCGM (no supplements, 2% FCS) were incubated with different concentrations of PDGF for 24 h prior to analysis of BrdU incorporation. Percent control represents comparison with the respective vehicle-treated group (set as 100%). Values are means ± SEM of *n* = 10–14 experiments. ^*^P < 0.05 vs. vehicle-treated group; one-way ANOVA plus Dunnett post hoc test.

### Inhibition of HO-1 protein expression increases cell death in HUASMC

**Figure 8:**
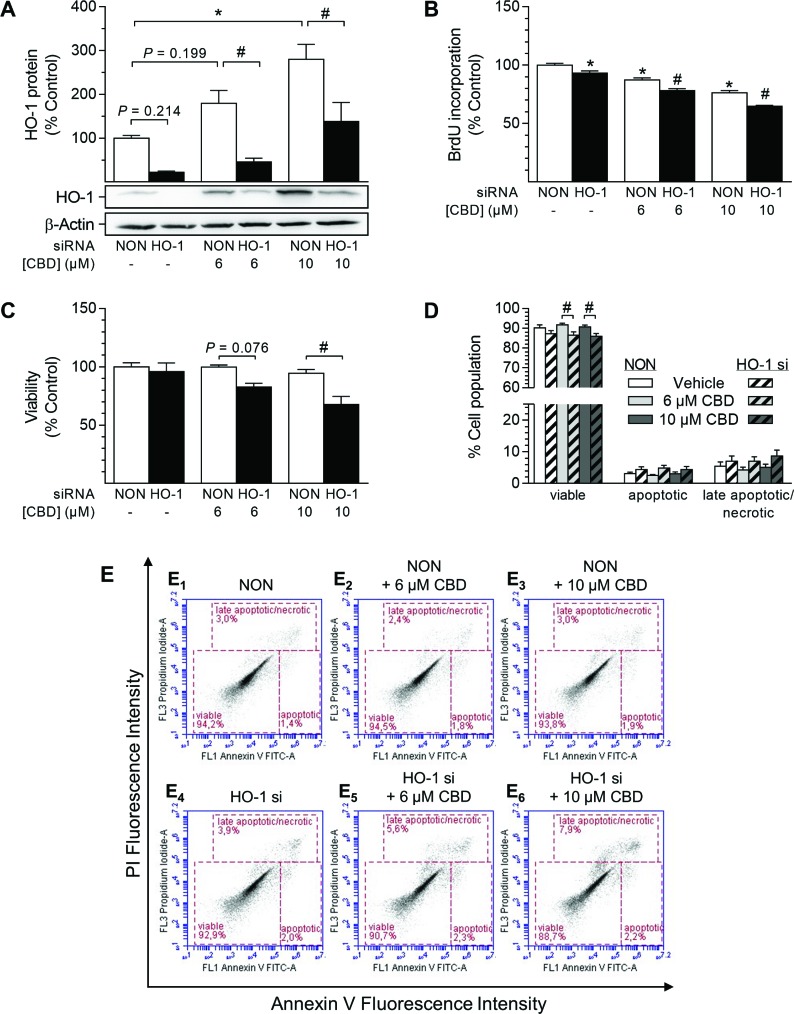
Impact of HO-1 siRNA on CBD-mediated anti-proliferative effects and viability of HUASMC Cells were transfected with HO-1-specific siRNA or non-targeting siRNA (NON) and further stimulated with CBD as indicated for 48 h (**A**, **C**, **D**) or 24 h (**B**). Following incubation, cells were analyzed for protein expression of HO-1 (A), BrdU incorporation (B), viability (C) or apoptosis (D). Representative images of flow cytometry analysis of apoptosis (D) are shown in (**E**). Protein expression values were normalized to β-actin. Percentage of control represents comparison with the respective vehicle-treated time-matched group (set as 100%). Western blot images are representative of the experiment. Values are means ± SEM of *n =* 4 (A), *n =* 11–12 (B), *n =* 3 (C) or *n =* 9 (D) experiments. ^*^P < 0.05 vs. time-matched NON-transfected vehicle control; ^#^*P* < 0.05 vs. concentration-matched NON-transfected sample; one-way ANOVA plus Šidák post hoc test.

To exclude possible unspecific effects of SnPPIX, additional siRNA approaches were performed to knock down HO-1 protein expression (Figure 8). In these experiments, induction of HO-1 protein expression by CBD at both 6 µM and 10 µM was significantly knocked down (Figure 8A). Likewise, basal HO-1 protein expression was reduced by 78% after transfection with HO-1 siRNA (Figure 8A). Similar to the results obtained with SnPPIX (Figure 7A), knockdown of HO-1 enhanced the anti-proliferative effect of CBD after a 24-h incubation period, although this was anti-proliferative per se (Figure 8B). In agreement with the results in Figure 7B, vital staining using trypan blue further showed an increase in cell death in HUASMC after knockdown of HO-1 in the presence of CBD at 6 µM and 10 µM (Figure 8C). However, the administration of HO-1 siRNA per se did not affect cell viability (Figure 8C). To further characterize the kind of cell death occurring in cells depleted of HO-1, a flow cytometry-based analysis of apoptosis was performed (Figure 8D). The results once more showed that CBD alone (6 µM and 10 µM) did not increase the number of apoptotic or late apoptotic/necrotic cells after 48 h of treatment (Figure 8D, 8E_1–3_). Conversely, the presence of HO-1 siRNA increased both apoptotic and late apoptotic/necrotic cell populations in CBD-treated HUASMC (Figure 8D, 8E_4–6_), thereby explaining the above-mentioned vital staining results (Figure 8C).

### CBD-mediated anti-migratory effects in HUASMC are not mediated via HO-1

**Figure 9:**
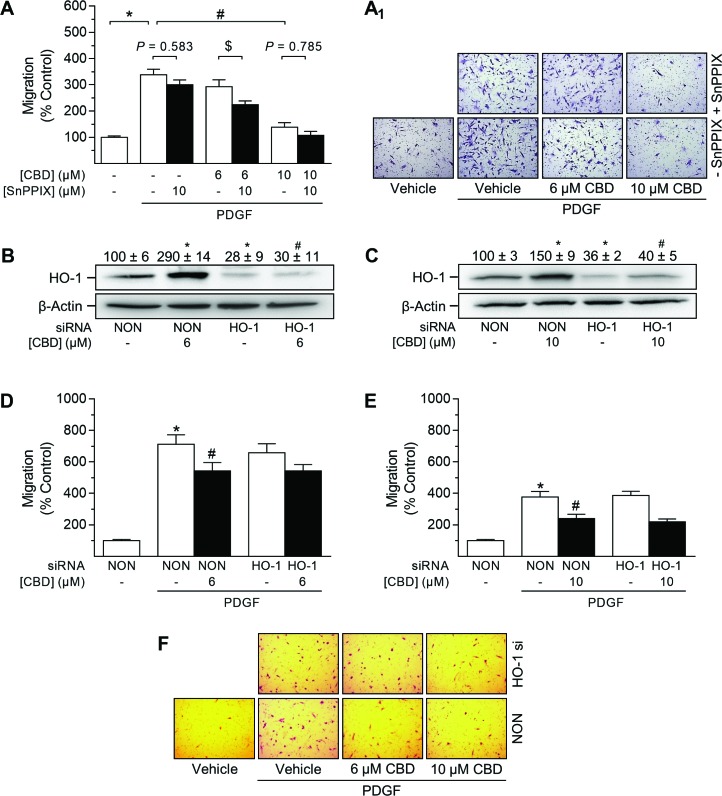
Impact of HO-1 siRNA on CBD-mediated anti-migratory effects in HUASMC Cells seeded in rSMCGM (no supplements, 2% FCS) were pre-incubated with 10 µM SnPPIX or vehicle prior to another 24-h co-incubation with vehicle or CBD at the indicated concentrations. Afterwards, viable cells were transferred to Falcon^®^ cell culture inserts. Migration towards PDGF (20 ng/ml) was run for 6 h (**A**). Cells were transfected with HO-1-specific siRNA or non-targeting siRNA (NON) and incubated in rSMCGM (no supplements, 2% FCS) for 24 h. For Western blot analysis, viable cells were transferred into 6-well plates and incubated with CBD or vehicle for 6 h prior to sample preparation (**B**, **C**). For migration, viable cells were transferred into Falcon^®^ cell culture inserts and incubated with CBD or vehicle for 1 h prior to the initiation of a 6-h migration period toward PDGF (20 ng/ml) or appropriate vehicle in the lower plate. Thereafter, inserts were prepared and migrated cells were counted in five randomly selected fields per insert (A, **D**, **E**). Representative images of migration analyses are shown in (**A**_**1**_, **F**). Protein expression values were normalized to β-actin. Percentage of control represents comparison with the respective vehicle-treated time-matched group (set as 100%). Western blot images are representative of each experiment. Values are means ± SEM of *n =* 15 (A), *n =* 3 (B, C), *n =* 28–36 (D) or *n =* 25 (E) experiments. ^*^P < 0.05 vs. PDGF-free vehicle control (A, D, E) or NON-transfected control cells (B, C); ^#^*P* < 0.05 vs. PDGF-treated vehicle control (A, D, E) or NON-transfected CBD-treated cells (B, C); ^$^*P* < 0.05 as indicated; one-way ANOVA plus Šidák post hoc test.

VSMC of the proliferative/synthetic phenotype are capable of enhanced migration [10]. Therefore, the effects of CBD on HUASMC migration were analyzed using a modified Boyden chamber assay (Figure 9). The role of HO-1 within this process was analyzed using both SnPPIX (Figure 9A, A_1_) and HO-1 siRNA (Figure 9B–9F). For successful migration towards the chemoattractant PDGF, cells were cultured in rSMCGM under serum-reduced conditions (2% FCS) and the withdrawal of other growth factors (no supplements) for the duration of the experiment. As shown in Figure 9A, PDGF significantly increased the migration of HUASMC to 330% vs. vehicle control (100%). Incubation with 10 µM CBD almost completely abolished this pro-migratory effect, resulting in 139% migration vs. vehicle control (100%). However, when tested at a concentration of 6 µM, the anti-migratory effect of CBD did not reach significance (Figure 9A). Similar to the results obtained from proliferation analysis (Figure 7A, 7D), inhibition of HO-1 activity by pre-incubation with SnPPIX enhanced CBD-mediated anti-migratory effects, and was slightly anti-migratory per se (Figure 9A, A_1_).

As shown in Figure 9B and 9C, both CBD-mediated and basal HO-1 protein expression were significantly knocked down by HO-1 siRNA. Evaluation of cell viability via vital staining revealed that HO-1 siRNA per se did not affect cell viability (99% ± 1.2% vs. non-targeting siRNA-transfected control [100% ± 1%]; means ± SEM of *n* = 8, Student’s two-tailed *t* test). Following 6 h of incubation, migration of HUASMC was significantly enhanced in the presence of PDGF (20 ng/ml) (Figure 9D, 9E). HO-1 siRNA per se had no effect on PDGF-induced HUASMC migration (Figure 9D, 9E). In contrast, CBD at 6 µM (Figure 9D) and 10 µM (Figure 9E) significantly inhibited the PDGF-induced migration of HUASMC transfected with non-targeting siRNA. However, knockdown of HO-1 protein expression did not alter the CBD-mediated inhibition of PDGF-induced migration (Figure 9D–9F).

## DISCUSSION

Inflammation-triggered proliferation and migration of VSMC are closely linked to atherosclerosis and restenosis [7, 13, 14], which are the underlying causes of many cardiovascular events [3]. Additionally, recruitment and activation of VSMC supports blood vessel formation during tumor development [8, 9]. Substances supporting the cellular anti-inflammatory capacity and/or inhibiting the abnormal proliferation and migration of VSMC may therefore meet a therapeutic need to control the progression of vascular and cancer diseases. The present study demonstrates that the phytocannabinoid CBD mediates the induction of the anti-oxidative enzyme HO-1 and the inhibition of growth factor-mediated proliferation/migration of VSMC, with both events occurring independently of each other.

Initial experiments demonstrated that two phytocannabinoids, CBD and THC, are able to induce the expression of HO-1 but not HO-2 in HUASMC, whereas the synthetic cannabinoids R(+)-methanandamide and JWH-133 were virtually inactive in this respect. Subsequent analyses were focused on CBD, which, in contrast to THC, is non-psychoactive, while exerting numerous beneficial pharmacological effects that have been reviewed in depth recently [38]. In our hands, CBD-mediated induction of HO-1 was present on both the mRNA and protein level and showed up in a time- and concentration-dependent manner. Concordantly, a comparable modulation of HO-1 in VSMC has been described for several other plant-derived substances [26, 27, 29, 30, 32, 33, 59]. Experimental approaches using antagonists targeting CB_1_, CB_2_, TRPV1 and GPR55 as well as a GPR55 agonist revealed no impact of these well-established cannabinoid-activated receptors on HO-1 induction by CBD. In line with our observations, CB_1_/CB_2_ receptor-independent actions of CBD have been described by others, including neuroprotection of cerebral artery occluded mice [60], prevention of chemotherapy-induced neuropathic pain in C57B1/6 mice [61] or impairment of U87 glioma cell migration [62]. On the other hand, although possessing low affinity towards CB_1_ and CB_2_ [63, 64], some CB-dependent effects of CBD have been reported in the literature, including CB_2_-dependent inhibition of murine macrophage chemotaxis [65] and a CB_2_-dependent anti-proliferative action on glioma cells [66]. Moreover, CBD has also been shown to cause endothelium-dependent vasorelaxation of pre-constricted human mesenteric arteries via the activation of CB_1_ and TRPV1, suggesting divergent actions of CBD dependent on the species and cell type analyzed [67]. Concerning GPR55, an antagonistic action of CBD on this receptor has been reported to induce mesenchymal stem cell migration via the activation of p42/44 mitogen-activated protein kinases [55]. However, in our hands, antagonistic effects of CBD towards GPR55 were excluded by use of the GPR55 agonist O-1602, which did not reverse the anti-proliferative and HO-1-inducing effects of CBD. Additionally, the CBD-mediated inhibition of proliferation was not enhanced by the GPR55 antagonist O-1918.

Instead, CBD-induced HO-1 expression was reversed by the glutathione precursor NAC, indicating the participation of ROS signaling in this process. In line with this finding, CBD was demonstrated to increase ROS generation in HUASMC in a NAC-reversible manner. As a matter of fact, an ambivalent role of CBD concerning oxidative stress is described in the literature, which is similar to its varying effects on receptors. Accordingly, the generation of ROS through the metabolism of CBD has been substantiated using mouse hepatic microsomes and electron spin resonance experiments [68]. Furthermore, CBD induces endoplasmic reticulum stress, followed by apoptosis of activated but not control hepatic stellate cells or primary hepatocytes [69]. On the other hand, CBD has been reported to reduce inflammation-induced endoplasmic reticulum stress in oligodendrocyte progenitor cells [48] and to counteract cisplatin-mediated oxidative stress-induced nephrotoxicity in mice [70]. Moreover, CBD attenuates lipopolysaccharide-induced vascular inflammation in the mouse brain [45] and reduces oxidative/nitrative stress in animal models of ischemia-reperfusion injury [50] and diabetic cardiomyopathy [51].

In the experiments described herein, HUASMC cultured in cSMCGM showed enhanced proliferation as compared to growth factor- and serum-starved cells. In line with this finding, Proudfoot *et al.* described the de-differentiation and enhanced proliferation of isolated human VSMC in culture, stating that isolated and cultured VSMC represent a disease-associated rather than a contractile/quiescent phenotype [71]. Moreover, modulation of VSMC phenotype is largely influenced by mitogens such as growth factors, including basic fibroblast growth factor and epidermal growth factor (reviewed in [10, 11, 72–74]), which were supplemented in the culture media used here. Thus, conditions conferring an amplified proliferation capacity of HUASMC were chosen as a suitable model to investigate the potential beneficial effects of CBD.

In the present study, functional analyses revealed CBD-mediated concentration-dependent inhibition of HUASMC proliferation and migration. Decreased proliferation apparently correlated with the induction of HO-1 by CBD, as it was not attenuated by substances targeting CB_1_, CB_2_, TRPV1 and GPR55. However, in our hands, the involvement of HO-1 in CBD-mediated anti-proliferative/anti-migratory effects was excluded by experiments demonstrating that both HO-1 siRNA as well as the inhibitor of HO-1 activity, SnPPIX, did not antagonize these responses. Moreover, experiments using NAC at a concentration sufficient to block ROS-mediated induction of HO-1 demonstrated that neither HO-1 nor ROS contributed to the CBD-mediated anti-proliferative effects in HUASMC. Similarly, it was shown that extracellular acidosis-mediated induction of HO-1 did not contribute to anti-proliferative/anti-migratory effects in wild-type and HO-1-null mouse aortic smooth muscle cells (SMC) [75].

Conversely, an anti-proliferative/anti-migratory function of HO-1 in VSMC has been reported in several other studies [25–32]. Accordingly, overexpression of human HO-1 in rat thoracic aorta SMC inhibits cell cycle progression and protects cells against hydrogen peroxide-mediated oxidative injury [76]. Concordantly, heme-induced ROS production and enhanced proliferation/migration of rat thoracic aorta SMC are increased by chemical inhibition of HO-1 [77]. Moreover, several substances conferring up-regulation of HO-1 have been shown to inhibit cytokine- or PDGF-induced proliferation and migration in SMC of different origin [25, 26, 29, 30]. Finally, bilirubin and carbon monoxide, both products of heme catabolism, have been demonstrated to reduce VSMC proliferation [78, 79].

The reason for these apparently contradictory data may lie in substance-dependent divergent mechanisms conferring anti-proliferative and anti-migratory effects in SMC. Thus, in line with the fact that both HO-1-dependent and -independent signaling may mediate the inhibition of SMC proliferation, different inducers of HO-1 have been revealed to elicit divergent effects in other cell types. Accordingly, Loboda *et al.* showed different mechanisms leading to the production of vascular endothelial growth factor (VEGF) in human microvascular endothelial cells by cobalt(II) chloride and cobalt protoporphyrin IX (CoPPIX), both being potent inducers of HO-1 [80]. Although both compounds induced HO-1 in these cells, the influence of cobalt(II) chloride on VEGF did not involve HO-1 and was dependent on the activation of hypoxia inducible factor 1α, while the effect of CoPPIX did not require hypoxia inducible factor 1α but rather relied on HO-1 [80]. Furthermore, it has been reported that genetic differences in GT repeat length influence the activity of the HO-1 promoter in human VSMC [81]. Therefore, species-dependent and donor-dependent genetic differences may affect the transcriptional response and thus strength of HO-1 activity, leading to divergent outcomes in different studies.

Indeed, the data of the present study indicate that inhibition or knockdown of HO-1 further enhanced the anti-proliferative/anti-migratory effects of CBD along with the induction of cell death and apoptosis, with the latter effect indicating a fundamental cytoprotective role of HO-1. Accordingly, a protective role of the HO-1 pathway against oxidative stress and apoptosis has also been shown in rat VSMC after treatment with the plant-derived triterpenoids maslinic acid and oleanolic acid [33, 59]. In line with our observations, Scott *et al.* reported alterations in the expression of several heat shock proteins (HSP), including HO-1, mediated by CBD-induced ROS generation with enhanced induction of apoptosis after co-incubation with HSP inhibitors in human glioblastoma cell lines [82]. Likewise, miR-24-mediated repression of HO-1 inhibited the proliferation and migration of human aortic SMC via the induction of apoptosis [83]. Furthermore, Wu *et al.* have reported that CBD induces a contrasting pro-apoptotic effect between freshly isolated and pre-cultured human monocytes. In that study, CBD markedly decreased the glutathione content in freshly isolated monocytes followed by the induction of apoptosis, whereas pre-cultured monocytes were insensitive against CBD-induced toxicity due to higher levels of glutathione and HO-1 [84]. Previously, the same group reported CBD-induced cytotoxicity in primary lymphocytes dependent on the oxidative stress-mediated induction of caspase-8 [85]. However, it should be noted that, in our experiments, VSMC treated with CBD alone did not undergo apoptosis, as analyzed by flow cytometry and vital staining, even though CBD intensely induced ROS production.

It is noteworthy that HO-1 upregulation as well as inhibition of proliferation and migration were significantly detectable at CBD concentrations of 6 µM and 10 µM. Although these concentrations are representative of the usual CBD concentrations used *in vitro*, ranging from 0.1–100 µM [48, 49, 66, 67, 69], they are indeed above the therapeutic plasma concentrations of the drug. Accordingly, mean CBD plasma levels of up to 0.036 µM (equates to 11.2 ng/ml) were detected following six weeks of oral treatment with CBD at a dose of 10 mg/kg/day (about 700 mg/day) [86]. Interestingly, in another recent study, a maximal CBD plasma concentration of 0.7 µM (equates to 221 ng/ml) was measured 3 h after co-administration of a single oral dose of CBD (800 mg; equates to 10–15 mg/kg) and a single intravenous dose of fentanyl (0.5 µg/kg) [87]. However, in view of the conditions and limitations of cell culture experiments, a direct correlation from *in vitro* to *in vivo* is in any case rather critical.

Clearly, more research is needed to understand the mode of action underlying the anti-proliferative and anti-migratory effects of CBD in HUASMC. However, to the best of our knowledge, the present study provides the first evidence to show an enhancement of HO-1 in VSMC via phytocannabinoid exposure as well as the first data indicating the beneficial effects of CBD on both proliferation and migration of VSMC. In conclusion, further studies addressing the vasculoprotective and anti-angiogenic properties of CBD are warranted.

## MATERIALS AND METHODS

### Reagents

CBD was bought from Biomol GmbH (Hamburg, Germany). JWH-133 and THC were bought from Bio-Techne GmbH (Wiesbaden, Germany) and Lipomed GmbH (Herne, Germany), respectively. R(+)-methanandamide, capsazepine and NAC were obtained from Sigma-Aldrich (Taufkirchen, Germany). AM251 and AM630 were obtained from Biozol (Eching, Germany). O-1602 and O-1918 were purchased from Tocris Bioscience (Bristol, UK) and Cayman Chemicals (Ann Arbor, Michigan, USA), respectively. SnPPIX was obtained from Enzo Life Sciences (Lörrach, Germany). The transfection reagent Lipofectamine™ RNAiMAX, OptiMEM and hPDGF-BB were purchased from Thermo Fisher Scientific Inc. (Schwerte, Germany). siRNA targeting HO-1 was purchased from Santa Cruz Biotechnology (Heidelberg, Germany; sc-35554). Negative control siRNA was from Qiagen (Hilden, Germany; cat. no. 1022076). Accutase cell detachment solution was obtained from Merck Chemicals GmbH (Darmstadt, Germany).

### Cell culture

Human umbilical artery smooth muscle cells (HUASMC) were purchased from Promocell (Heidelberg, Germany). Expansion, cell cultivation and most experiments were performed using complete smooth muscle cell growth medium (cSMCGM) supplemented with 5% FCS, 0.5 ng/ml epidermal growth factor, 2 ng/ml basic fibroblast growth factor and 5 ng/ml insulin (all from Promocell). In experiments using PDGF, cells were seeded and stimulated in reduced SMCGM (rSMCGM: no supplements, 2% FCS). Cells were cryopreserved in liquid nitrogen using cryo-SFM freezing medium (Promocell). HUASMC at passages 2 to 6 were grown in a humidified incubator at 37° C and 5% CO_2_ and used for experiments. HUASMC were seeded in cell culture well plates at following densities, if not otherwise indicated: 6-well, 1.5 × 10^5^ cells per well; 12-well, 6 × 10^4^ cells per well; 24-well, 3.3 × 10^4^ cells per well; 96-well, 5 × 10^3^ cells per well. Cells were allowed to adhere and grow for 24 h prior to medium change and stimulation with test substances. A 1-h pre-incubation was performed in co-incubation experiments using NAC, SnPPIX and substances targeting CB_1_ (AM251), CB_2_ (AM630), TRPV1 (capsazepine) or GPR55 (O-1918, O-1602). Most test substances were dissolved in DMSO or in ethanol and further diluted in cSMCGM or rSMCGM. Final concentrations in incubates were as follows: DMSO, 0.01% (v/v) (for AM251, AM630, capsazepine, O-1602 and O-1918); ethanol, 0.033% (v/v) (for CBD and THC) and 0.1% (v/v) (for JWH-133 and R(+)-methanandamide). SnPPIX was diluted in 1 M NaOH resulting in a final NaOH concentration of 0.04% (v/v) in incubates. NAC was diluted in PBS resulting in a final PBS concentration of 0.6% (v/v) in incubates. PDGF was diluted in 0.1 M acetic acid/0.1% (m/v) bovine serum albumin resulting in a final solvent concentration of 0.02% in incubates. Samples and controls of the respective experiments were adjusted to equal solvent concentrations in incubates.

### siRNA transfection

Knockdown of HO-1 protein expression was performed using Lipofectamine™ RNAiMAX transfection reagent in a reverse procedure according to the manufacturer’s instructions (Thermo Fisher Scientific Inc.). For one well of a 6-well plate, transfection complexes were generated by mixing 45 pmol siRNA with 5 µl Lipofectamine™ RNAiMAX in OptiMEM to a final volume of 500 µl. The solutions were mixed thoroughly and incubated for 20–30 min at room temperature to form transfection complexes. HUASMC (1.5 × 10^5^cells/2.5 ml SMCGM per well) were seeded in 6-well plates already containing transfection complexes. Control cells were transfected with a non-targeting siRNA. Experimental settings were adapted for biochemical assays in 96-well plates. The final concentration of siRNA was 15 nM. After 24 h, transfection medium was replaced with fresh SMCGM and cells were treated as stated above or used for migration experiments as described below.

### Vital staining

Membrane integrity was analyzed as a parameter for cell viability using vital staining with trypan blue. In this assay, discrimination of viable and dead cells occurs via exclusion of the stain by intact cell membranes of viable cells. Damaged cells or cells undergoing apoptosis lose membrane integrity and therefore incorporate the stain. Briefly, cells seeded in 6-well or 12-well plates were incubated with test substances. Afterwards, cells were washed with PBS and collected by trypsinization. Samples were centrifuged for 5 min at 200 × *g* and carefully resuspended in cSMCGM. Analysis was performed on samples diluted 1:2 [v/v] with 0.4% trypan blue stain (Thermo Fisher Scientific Inc.) in a Luna-II™ automated cell counter (Biozym Scientific, Hessisch Oldendorf, Germany) using a cell diameter range from 10–30 µm.

### Cell proliferation assay

The impact of test substances on proliferation of HUASMC was quantified using a BrdU Cell Proliferation ELISA kit (Abcam, Cambridge, UK). BrdU is incorporated into the newly synthesized DNA of dividing cells. Thus, BrdU labeling of cells directly correlates to the rate of DNA synthesis during the S phase of the cell cycle. Briefly, cells seeded in 96-well plates were challenged with test substances or the appropriate vehicle for the indicated times. BrdU reagent was added 24 h prior to analysis, except for 6-h experiments where BrdU was added simultaneously with test substances. After incubation, the supernatant was removed, and the assay was performed according to the manufacturer’s instructions. Absorption was measured at 450/550 nm using an ELISA plate reader. Cells that did not receive BrdU reagent served as the blank. Each experiment consisted of four repeats per sample.

### Migration assay

The effect of test substances on migration of HUASMC was determined using a modified Boyden chamber assay. This assay monitors cellular motility as migration through Falcon^®^ cell culture inserts (8 µm pore size; Corning Inc., Corning, NY, USA) towards a chemoattractant in the companion plate. In order to investigate the role of HO-1 in HUASMC migration, two different experimental settings were used. First, cells seeded in rSMCGM were subjected to treatment with the HO-1 activity inhibitor SnPPIX and test substances as stated above (see section: Cell culture). After incubation, cells were counted, vital HUASMC (5 × 10^4^ cells/0.5 ml rSMCGM) were seeded in Falcon^®^ cell culture inserts and migration was initiated. Second, in rSMCGM reverse-transfected HUASMC (see section: siRNA transfection) were counted, vital HUASMC (3 × 10^4^ cells/0.5 ml rSMCGM) were seeded in Falcon^®^ cell culture inserts and incubated with CBD (6 µM and 10 µM) or vehicle for 60 min at 37° C and 5% CO_2_ before the initiation of migration. In both setups, migration was initiated by the addition of 500 µl rSMCGM containing PDGF (20 ng/ml) or vehicle to the companion plate. Cells were further incubated for 6 h. Non-migrated cells on the upper surface of the insert were removed with a cotton swab. Cells on the lower surface of the inserts were fixed and stained with Diff Quick stain (Labor + Technik Eberhard Lehmann GmbH, Berlin, Germany) according to the manufacturer’s instructions. After complete drying, inserts were photographed using a 10× objective and digital microscope camera (AxioCam ERc 5 s; Zeiss, Oberkochen, Germany). Five randomly selected fields per insert were counted.

### Flow cytometric detection of oxidative stress

Analysis of oxidative stress in HUASMC was performed using the CellROX™ Green Flow Cytometry Assay kit according to the manufacturer’s instructions (Thermo Fisher Scientific Inc.). The CellROX™ Green reagent is a cell-permeable probe that, when oxidized in the presence of ROS, binds to DNA and exhibits a strong fluorogenic signal with absorption/emission maxima of 508/525 nm. Co-staining with SYTOX™ Red dead cell stain allows for the distinction between oxidative stressed, non-stressed and dead cells. Briefly, cells seeded in 12-well plates were pre-incubated with NAC followed by a co-incubation period with CBD for another 4 h. CellROX™ Green reagent (final concentration in incubates: 500 nM) was added 1 h before sample preparation. Then, floating cells were collected and combined with the adherent cells that were harvested by detachment with 300 µl of Accutase solution, according to the manufacturer’s instructions. Cells were pelleted by centrifugation (200 × *g*, 5 min) and carefully resuspended in 200 µl cSMCGM. Finally, SYTOX™ Red dead cell stain (final concentration in incubates: 5 nM) was added and cells were incubated in the dark for 15 min at room temperature. Subsequently, 10^4^ cells per sample were analyzed using an AccuriC6™ flow cytometer (BD Biosciences, Heidelberg, Germany). Appropriate gating of cell populations and compensation was ensured by the analysis of unstained and single-stained cells, respectively. HUASMC that were treated with the ROS inducer TBHP (250 µM) for 1 h prior to sample preparation served as the positive control. Negative control cells were pre-incubated with NAC (0.5 mM) prior to stimulation with TBHP.

### Flow cytometric detection of apoptosis

Detection of apoptosis was accomplished by flow cytometry using a fluorescein isothiocyanate (FITC)-Annexin V (AV) Apoptosis Detection kit according to the manufacturer’s instructions (BD Biosciences). During apoptosis, cells undergo certain morphological changes, such as loss of plasma membrane asymmetry, cell shrinkage and condensation of the cytoplasm and nucleus. AV is a phospholipid-binding protein with high affinity for phosphatidyl serine, which is exposed on the outer side of apoptotic but not viable cells. Both apoptosis and necrosis lead to the loss of membrane integrity in cells. Therefore, co-staining with propidium iodide (PI), a membrane-impermeable DNA dye, allows for the distinction between early and late apoptotic cells. Cells that are considered viable are negative for both FITC-AV and PI; cells in early apoptosis are positive for FITC-AV but negative for PI. However, due to the loss of membrane integrity and binding of both FITC-AV and PI, there is no precise differentiation between late apoptotic and necrotic cell populations in this assay.

Briefly, cells seeded in 12-well plates were incubated with the test substances. For sample preparation, floating cells were collected and combined with the adherent cells that were harvested by detachment with 300 µl of Accutase solution, according to the manufacturer’s instructions. Cells were pelleted by centrifugation (400 × *g*, 4° C, 5 min) and washed twice in 500 µl PBS. Afterwards, cells were carefully resuspended in a solution consisting of 200 µl 1X AV-binding buffer, 2 µl FITC-AV and 1 µl PI solution. Following 15 min of incubation in the dark, 10^4^ cells per sample were analyzed using an AccuriC6™ flow cytometer (BD Biosciences). Appropriate gating of cell populations and compensation was ensured by the analysis of unstained and single-stained cells, respectively. HUASMC that were fixed with ethanol prior to incubation with AV and PI served as the staining control.

### Quantitative RT-PCR analysis

For the quantification of mRNA expression, cells were seeded in 24-well plates and treated as described above. After exposure to the test substances or vehicles for the indicated times, total RNA was isolated using the RNeasy Mini kit. Expression levels of HO-1, HO-2 and β-actin mRNA were determined by quantitative RT-PCR using the TaqMan^®^ RNA-to-C_T_™ 1-Step kit (Thermo Fisher Scientific Inc.). Primers and probes for human β-actin, HO-1 and HO-2 were Gene Expression Assay™ products (Thermo Fisher Scientific Inc.). All experiments were performed according to the manufacturer’s instructions. HO-1 and HO-2 mRNA levels were normalized to β-actin and samples were compared to the appropriate vehicle controls.

### Western blot analysis

For the preparation of whole cell lysates, floating and adherent cells were collected and resuspended in 50 µl of sample buffer (62.5 mM Tris/HCl, 2% [v/v] sodium dodecyl sulfate (SDS), 10% [v/v] glycerol). Cells were then lysed by sonication, heated for 5 min at 95° C and centrifuged at 14,000 rpm for 5 min at 4° C. Supernatants were analyzed to determine the total protein concentration using a Pierce^®^ bicinchoninic acid (BCA) protein assay kit (Thermo Fisher Scientific Inc.) according to the manufacturer’s protocol. Samples containing 50 µg of protein and 5% (v/v) β-mercaptoethanol were separated by 12% SDS-polyacrylamide gel electrophoresis and transferred to nitrocellulose membranes by electroblotting (75 min) using a semi-dry transfer system, according to the manufacturer’s instruction (Bio-Rad, Munich, Germany). Membranes were blocked for 1 h in 5% Blotting Grade Blocker (Bio-Rad) in Tris-buffered saline/Tween^®^ 20 (TBS-T) before incubation with specific primary antibodies at 4° C overnight. Primary antibodies were bought from Enzo Life Sciences GmbH (HO-1, HO-2), Sigma-Aldrich (β-actin), Thermo Fisher Scientific Inc. (TRPV1, GPR55) and Santa Cruz Biotechnology (CB_1_, CB_2_), respectively. Afterwards, membranes were washed with TBS-T and probed with appropriate horseradish peroxidase-linked secondary antibodies. Secondary antibodies directed against mouse or rabbit IgG were purchased from Cell Signaling Technology Europe (Leiden, The Netherlands). Antibody binding was visualized by chemiluminescence and quantified by densitometric analysis using Quantity One 1-D Analysis Software (Bio-Rad). After analysis, membranes were stripped (200 mM glycine, 500 mM sodium chloride, pH 2.5) and reprobed. Expression of proteins was normalized to β-actin and compared to the appropriate vehicle controls.

### Statistics

The measurement values of cell proliferation were analyzed for statistical outliers in each experiment using the Nalimov test. Statistical analysis was performed using GraphPad Prism 6.01 (GraphPad Software, San Diego, CA, USA). One-way ANOVA plus the Dunnett post hoc test was used for the comparison of samples to vehicle control. Comparisons among selected groups were carried out by Student’s two-tailed *t* test (for kinetics) or multiple comparison tests using one-way ANOVA plus the Šidák post hoc test. All values are presented as means ± standard error of the mean (SEM). A *P* value *P* < 0.05 was considered significant.

## Abbreviations

AM251: 1-(2,4-dichlorophenyl)-5-(4-iodophenyl)-4-methyl-N-piperidin-1-ylpyrazole-3-carboxamide
AM630: [6-iodo-2-methyl-1-(2-morpholin-4-ylethyl)indol-3-yl]-(4-methoxyphenyl)methanone
AV: annexin V
BrdU: bromodeoxyuridine
CB_1_: cannabinoid receptor type 1
CB_2_: cannabinoid receptor type 2
CBD: cannabidiol
CoPPIX: cobalt protoporphyrin IX
FCS: fetal calf serum
FITC: fluorescein isothiocyanate
GPR55: G protein-coupled receptor 55
HO-1: heme oxygenase-1
HO-2: heme oxygenase-2
HSP: heat shock protein
HUASMC: human umbilical artery smooth muscle cells
JWH-133: (6aR,10aR)-6,6,9-trimethyl-3-(2-methylpentan-2-yl)-6a,7,10,10a-tetrahydrobenzo[c]chromene
MFI: mean fluorescence intensity
NAC: N-acetylcysteine
O-1602: 5-methyl-4-[(1R,6R)-3-methyl-6-prop-1-en-2-ylcyclohex-2-en-1-yl]benzene-1,3-diol
O-1918: 1,3-dimethoxy-5-methyl-2-[(1R,6R)-3-methyl-6-prop-1-en-2-ylcyclohex-2-en-1-yl]benzene
PBS: phosphate-buffered saline
PI: propidium iodide
ROS: reactive oxygen species
RT-PCR: reverse transcriptase polymerase chain reaction
SDS: sodium dodecyl sulfate
siRNA: small-interfering RNA
SMC: smooth muscle cells
SMCGM: smooth muscle cell growth medium
SnPPIX: tin protoporphyrin IX
TBHP: tert-butyl hydroperoxide (2-hydroperoxy-2-methylpropane)
THC: Δ^9^-tetrahydrocannabinol
TRPV1: transient receptor potential vanilloid 1
VEGF: vascular endothelial growth factor
VSCM: vascular smooth muscle cells
